# Sir Edwin Saunders, F.R.C.S.

**Published:** 1901-06

**Authors:** 


### SIR EDWIN SAUNDERS, F.R.C.S.

It is with much regret that we have to record the death on March 15 of Sir Edwin Saunders, at "Fairlawn," Wimbledon Common. Born in 1814, his long life closes with a century which must be memorable in the history of our profession; to the making of that history, so far as it concerns dentistry, Sir Edwin played a considerable part. When the first efforts were made towards united action in 1856, Sir Edwin Saunders was among those who petitioned the Royal College of Surgeons of England to give a status to practising dentists by means of examination and diploma. And though this end was obtained later, it was not until the College had received a special sanction from Parliament that the first dental qualification was instituted.

For the mutual interchange of experience in dental matters the Odontological Society was formed. The name of the Society was suggested by Sir Edwin Saunders, and the preliminaries were arranged at a meeting at his house in George Street, Hanover Square. It was in his house also that some of the earliest administrations of ether took place.

Sir Edwin was twice elected President of the Odontological Society, in 1864 and 1879, and his portrait, painted by subscriptions of his professional friends, was presented to the Society.

As a stepping-stone to the dental diploma, the first dental hospital in Soho Square was formed in 1859, and the school in 1860, and ten years later, when increased accommodation was urgently required, it was chiefly to Sir Edwin Saunders's indefatigable perseverance that a site was secured and the hospital removed from its retired and little-known position to the more advantageous site in Leicester Square. By the generosity of Sir Edwin and other friends of the hospital it started on its new career free of debt; the later additions to the building being also largely due to Sir Edwin's help. As an appropriate testimonial a sum was raised by the profession which took the form of a Saunders Scholarship.

At the second meeting in London of the British Dental Association, in 1886, Sir Edwin Saunders was President. He was elected President of the Metropolitan Counties Branch of the British Medical Association in 1881, the honor conferred by our *confrères* being much appreciated by the dental profession, and in the same year, at the meeting in London of the International Medical Congress, Sir Edwin presided over the Dental Section.

For more than forty years Sir Edwin Saunders was Dental Surgeon to her late Majesty Queen Victoria and the late Prince Consort. Very many others of the Royal Family were also under his care. Sir Edwin possessed many tokens of her late Majesty's appreciation, and in 1883 he received the honor of knighthood.

Sir Edwin was many-sided in his tastes. He was president of the Chrysanthemum Society and also an energetic supporter of the Botanical Gardens. Many members of the profession will remember his beautiful house at Wimbledon, and its hospitality. On the occasion of his golden wedding an illuminated album, in which were inscribed the names of his professional friends, was presented to him, Lady Saunders receiving a diamond brooch at the same time.

Sir Edwin Saunders was buried at Putney Vale Cemetery, on March 20, the funeral being attended by many of his friends and admirers.—*The Dental Record.*

